# Systematic Comparison of Epidemic and Non-Epidemic Carbapenem Resistant *Klebsiella pneumoniae* Strains

**DOI:** 10.3389/fcimb.2021.599924

**Published:** 2021-02-23

**Authors:** Katariina Koskinen, Reetta Penttinen, Anni-Maria Örmälä-Odegrip, Christian G. Giske, Tarmo Ketola, Matti Jalasvuori

**Affiliations:** ^1^Department of Biological and Environmental Science, Nanoscience Center, University of Jyväskylä, Jyväskylä, Finland; ^2^Department of Biology, University of Turku, Turku, Finland; ^3^Division of Clinical Microbiology, Department of Laboratory Medicine, Karolinska Institutet, Stockholm, Sweden; ^4^Department of Clinical Microbiology, Karolinska University Hospital, Stockholm, Sweden

**Keywords:** XDR *Klebsiella pneumoniae*, extended-spectrum beta-lactamase, epidemic, antibiotic resistance, virulence

## Abstract

Over the past few decades, extensively drug resistant (XDR) resistant *Klebsiella pneumoniae* has become a notable burden to healthcare all over the world. Especially carbapenemase-producing strains are problematic due to their capability to withstand even last resort antibiotics. Some sequence types (STs) of *K. pneumoniae* are significantly more prevalent in hospital settings in comparison to other equally resistant strains. This provokes the question whether or not there are phenotypic characteristics that may render certain *K. pneumoniae* more suitable for epidemic dispersal between patients, hospitals, and different environments. In this study, we selected seven epidemic and non-epidemic carbapenem resistant *K. pneumoniae* isolates for extensive systematic characterization for phenotypic and genotypic qualities in order to identify potential factors that precede or emerge from epidemic successfulness. Studied characteristics include growth rates and densities in different conditions (media, temperature, pH, resource levels), tolerance to alcohol and drought, inhibition between strains, ability to compensate pH, as well as various genomic features. Overall, there are clear differences between isolates, yet, only drought tolerance was found to notably associate with non-epidemic *K. pneumoniae* strains. We further report a preliminary study on the potential to control *K. pneumoniae* ST11 with an antimicrobial component produced by a non-epidemic *K. pneumoniae*. This component initially restricts bacterial growth, but stable resistance develops rapidly *in vitro*.

## Introduction

*Klebsiella pneumoniae* is a Gram-negative bacillus causing opportunistic infections outside of the gastrointestinal tract (Podschun and Ullman, 1998). Common conditions include pneumonia, urinary tract infections, wound infections, and less often liver abscess, meningitis, and septicemia. Some of the strains circulating in clinical settings are also showing increasingly virulent phenotypes (Pomakova et al., 2012; Shon et al., 2013). These strains are often characterized by hypermucoviscosity when cultivated on agar plates and they are more resilient against killing by serum or phagocytosis (Catalan-Najera et al., 2017). Moreover, extensively drug-resistant (XDR) among *K. pneumoniae* is increasing very rapidly compared to many other priority pathogens (World Health Organization, 2014). In particular, infections caused by *K. pneumoniae* strains which have developed resistance against newer generations of β-lactams, such as carbapenems, can be hazardous and often life-threatening. These carbapenemase genes are found to be abundant in bacteria originating from hospital environments although there are notable regional differences. Yet, sequence typing of the pathogens indicate that resistant *K. pneumoniae* strains can also disseminate globally between hospitals.

WHO has classified carbapenemase-producing *K. pneumoniae* as an urgent threat (World Health Organization, 2017). Often, *K. pneumoniae* isolates are typed by utilizing partial sequences from seven housekeeping genes. These genes are part of the core genome and hence unlikely to be horizontally transferred between different *K. pneumoniae* strains. As such, sequence typing provides a rudimentary approach to identify genetic similarity among isolates of different sources of origin. It appears that certain sequence types (STs) have been more successful in dispersing between hospitals compared to other equally resistant strains. NDM-1 metallo-β-lactamase producing ST11 and 14 have been noted to be responsible of epidemics in various countries (Yong et al., 2009; Pitout et al., 2015; Samuelsen et al., 2017). *Klebsiella pneumoniae* carbapenemase (KPC) producing ST258 have even been referred as hyperepidemic clone (Bowers et al., 2015) and its epidemic potential has been further investigated in several meta-analyses (Dautzenberg et al., 2016). ST512, a single-locus variant of ST258, is also highly associated in epidemics globally (Conte et al., 2016). ST147 is also hazardous with numerous virulence and resistance genes (Turton et al., 2018). Despite of the notion that certain STs appear to be more prone for inter-hospital dispersal, it is still unclear what qualities alongside of pathogenicity, if any, may be responsible for this epidemic success. Majority of the surveillance attempts focus on resistance profiles and genotypic features (Giske et al., 2012). Genomic data also accumulates rapidly as whole genome sequences of many strains have become available (Holt et al., 2015). Yet, the phenotypic characteristics of differently successful *K. pneumoniae* STs are rarely studied in detail, or the phenotypic analysis focus on specific traits such as hypermucoviscousity (Catalan-Najera et al., 2017). Comparison of the phenotypes of epidemic and non-epidemic strains could potentially reveal meaningful interactions between bacteria and their environment that contribute to the epidemic spread of XDR strains.

In this study, we selected 14 carbapenem resistant *K. pneumoniae* strains isolated from patients hospitalized in USA, Sweden, UK, Greece, or India (Kitchel et al., 2009; Samuelsen et al., 2009; Kitchel et al., 2010; Samuelsen et al., 2011; Vading et al., 2011; Giske et al., 2012; Hasan et al., 2014). Five of these STs are continuously being detected in hospitals in multiple countries and can be considered as epidemiologically successful or epidemic (**Table 1**). The rest of the STs have made only seldom appearances and have rarely if at all dispersed to other hospitals and may hence be considered as non-epidemic STs. Here, we systematically determined and measured potentially relevant characteristics for these strains in order to reveal differences that may correlate and perhaps partly explain the epidemic success.

**Table 1:** K. *pneumoniae* isolates used in the study.

	Isolate	ST from database	Isolation location	No. of CRISPR loci	No. of prophage regions	Reference	Virulence genes	Capsule types	Beta-lactamase
NKP01	1534	37	USA	0	10	Kitchel et al., 2009	*mrk*	K15K17K50K51K52	blaTEM-1B, blaKPC-2, blaSHV-11
NKP02	10924	334	USA	0	4	Kitchel et al., 2009	*mrK*	N/A	blaKPC-3, blaOKP-B-4, blaOXA-9, blaTEM-1A
EKP03	70165	14	USA	3	10	Kitchel et al., 2009	*irp, fyu, ybt, kfu, mrk*	K2	blaTEM-1A, blaSHV-28, blaKPC-3
EKP05	70708	258	USA	0	7	Kitchel et al., 2009	*mrk*	N/A (K15K17K50K51K52)	blaOXA-9, blaKPC-3, blaSHV-12
EKP08	2008025	11	USA	0	11	Kitchel et al., 2009	*mrk*	K13	blaKPC-2, blaSHV-11
EKP10	AO-8053	512	Sweden (Israel*)	0	10	Samuelsen et al., 2009	*mrk*	N/A	blaTEM-1A, blaOXA-9, blaSHV-11, blaKPC-3
EKP11	AO-15200	147	Sweden (Greece*)	2	6	Samuelsen et al., 2011	*mrk*	K64,K14	blaSHV-11, blaVIM-1
NKP18	VPKP389	36	Athens, Greece	1	10	Hasan et al., 2014	*irp, mrk, fyu, ybt*	k27	blaSHV-129, blaVIM-26
NKP20	VPKP229	17	Athens, Greece	0	8	Hasan et al., 2014	*irp, mrk, ybt*	k25	blaSHV-129, blaVIM-1
EKP22	N6	14	UK	2	6	Giske et al., 2012	*mrk, ybt, kfu*	k2	blaCTX-M-15, blaSHV-11, blaTEM-1A, blaOXA-1, blaNDM-1, blaOXA-9
EKP24	ED502873	11	Sweden	0	6	Giske et al., 2012	*irp, mrk, fyu, ybt*	N/A (K15K17K50K51K52)	blaSHV-11, blaCTX-M-15, blaNDM-1
NKP25	N12	231	UK	1	4	Giske et al., 2012	*kfu, mrk*	K51	blaSHV-1, blaTEM-1B, blaNDM-1, blaOXA-1
NKP28	B357	43	UK	1	8	Giske et al., 2012	*mrk, kfu*	K30	blaCTX-M-15, blaDHA-1, blaCMY-6, blaSHV-11, blaOXA-9, blaNDM-1, blaTEM-1A
NKP30	IR34	624	Chennai, India	1	3	Giske et al., 2012	*mrk*	K12, K29	blaTEM-1B, blaDHA-1, blaNDM-1, blaSHV-36, blaCTX-M-15, blaOXA-1

*Isolates associated with import from the country.

## Materials and Methods

### Strains

All 14 studied *Klebsiella pneumoniae* strains (see **Table 1**) were Illumina sequenced at Karolinska Institutet, Sweden. Genome sequences can be found from GenBank under BioProject id PRJNA680903. Sequences of EKP24 and NKP2 were also PacBio sequenced in University of Helsinki, Finland. Sequenced genomes were annotated by Rapid Annotation using Subsystem Technology (RAST, https://rast.nmpdr.org) and both secure and potential protein coding genes were mapped and the distribution of protein families were compared between the strains. Resistance genes were identified with ResFinder (https://cge.cbs.dtu.dk/services/ResFinder/), prophages with Prophage Finder (https://omictools.com/prophage-finder-tool), and CRISPR-regions with CRISPR-finder (https://crispr.i2bc.paris-saclay.fr). An algorithm was written to identify unannotated short open reading frames (ORFs) from the genome files (**Supplementary File 1**). The algorithm scans the genome for ORFs that have a potential ribosome binding site upstream of the start codon and is not overlapping with annotated genes.

### Growth Experiments

Growth densities to each strain were measured in temperature of +37°C and room temperature with 230 rpm shaking or without shaking. Cells were grown overnight in 5 ml of LB media (+37°C, 230 rpm) and then transferred into 5 ml of fresh LB media in 1:5,000 ratio. Fresh cultures were grown in experimental settings of +37°C and 230 rpm or RT and 0 rpm for 20 h and growth densities were calculated as colony forming units (cfu)/ml. As a standard initial liquid culture for the growth curve experiments all the strains were cultured in 5 ml of 100% LB, +37°C, and 230 rpm overnight and then transferred into experimental settings. In order to test the effect of shaking, the initiating cultures were prepared then transferred into 5 ml of LB in 1:100 ratio and grown in experimental settings of +37°C and 230 rpm and +37°C and 0 rpm. In growth curve experiments the effect of different media and varying concentrations and compositions of nutrients on growth was determined for each strain. One hundred percent LB was used throughout the experiments unless mentioned otherwise. Growth curves were measured in 10 and 1% LB, 100% BHI, and 100% of pure DMEM by diluting the initial culture in 1:100. Growth curves were measured at +37°C, 595 nm wavelength with Multiscan FC (Thermo Scientific) for 20 h in 5 min intervals and maximum growth and average growth rate were calculated.

### Survival in Acidic pH and Compensation Capacity

Bacterial cells’ ability to tolerate acidic surrounding pH and capability to compensate it by metabolism was measured for each strain in pH 3–7. Initial cultures were grown in 5 ml LB pH 7 at +37°C and 210 rpm overnight. Each strain was then transferred into 5 ml LB of either pH 3, pH 4, pH 5, pH 6, or pH 7 in 1:100 ratio and cultured in +37°C and 210 rpm. In pH 5–7 cultures were grown for 90 h and growth densities were calculated by plating in 16, 24, and 90 h. Then 1.5 ml of culture was filtered through 0.2 μm and supernatant pH was measured with Basic pH Meter (Denver Instruments) in 24 and 90 h. Cultures in pH 3–4 were shortened into 24 h experiment and growth densities were calculated in 16 and 24 h and supernatant pH was measured in 24 h. Growth curves were measured by diluting the initial pH 7 cultures in 1:100 ratio into LB pH of 3–7 and growth curves were measured at +37°C, 595 nm wavelength for 20 h in 5 min intervals.

### Cross-Strain Interactions

In aim to study the dynamics all the strains were cultured separately, and their metabolic products secreted into surroundings were tested against other strains in cross-strain inhibition experiments. Cross-strain interactions were tested by collecting the media after overnight culturing at +37°C and 210 rpm. Overnight cultures were centrifuged first with 7,000 × g for 4 min, and the supernatant was centrifuged again with 10,000 × g for 1 min. Each strain was cross-plated with all the supernatants (overnight, +37°C), and the inhibition of the growth of each strain was observed.

### Supernatant Inhibition and Prophages

A 4-week evolutionary experiment was designed to study appearance, persistence, and reversibility of putative colicin E3 resistance in sensitive EKP24 strain. For the first 2 weeks EKP24 was cultured with (n = 5) and without (n = 5) colicin E3 in 10% LB media supplemented with either NKP2 (containing colicin E3) or EKP24 (not containing colicin E3) supernatant filtrate (0.2 μm) in 1:4 ratio. Ten percent LB media was supplemented with 25 μg/ml of kanamycin and 150 μg/ml of ampicillin. After 2 weeks EKP24 cultured with the presence of colicin E3 were divided into two sets of samples (both n = 5). Other set was continued with colicin E3 exposure as described earlier. In the other set of samples, colicin E3 containing supernatant was replaced with EKP24 supernatant. Cultures were refreshed in 1:100 ratio three times and samples stored once a week with glycerol at −80°C. Development and persistence of colicin E3 resistance was determined by plating. Samples were taken at the beginning of the experiment, before division of colicin E3 exposed EKP24, and at the end of the experiment and were used for DNA extraction. DNA was isolated with DNeasy Blood & Tissue Kit (Qiagen) and sequenced with Illumina HiSeq. The observed reads were mapped to original PacBio-sequenced genome of NKP2 (described above) in order to detect the genetic variants. The variants developed under the exposure of colicin E3 were identified by filtering out those variants that were already present in the beginning of the experiment. The genetic analysis was performed with CLC Genomics Workbench v11 (Qiagen).

Interactions between the putative colicin E3 producing NKP2 and susceptible EKP24 bacterial cells were also observed with confocal microscopy. Then 200 μl of 1% LB-agar was placed into a chamber of eight-chambered ibidi^®^ ibiTreat μ-Slide (Ibidi GmbH) covered with CID lid for µ-dishes (Ibidi GmbH), and 3 μl of NKP2 and EKP24 were injected under the agar onto opposite sides. Encounter of the two strains was visualized with Nikon AR1 laser scanning confocal microscope with 60× water immersion objective and using Galvano scanner.

Production of putative colicin E3 was further studied by growing NKP2 strain in different medias. NKP2 was grown in LB concentrations of 100, 10, and 1%, LB without tryptone, 100% Shieh (Song et al., 1988) and in 100% DMEM in +37°C and 200–230 rpm overnight. NKP2 cultures were filtered through 0.2 μm filter and colicin E3 presence was determined by plating the supernatant with susceptible EKP24 strain.

### Alcohol Exposure

All studied strains were exposed to multiple concentrations of ethanol (20, 50, 75, and 90%) and their ability to survive the exposure were measured with spectroscopy. In 30 s exposure experiment all the strains were first grown in 5 ml of LB (+37°C, 230 rpm, overnight) and then transferred in 1:10 ratio into fresh LB and left to grow overnight at +37°C on 96-well plate 100 μl per well to form biofilm. On the following day media was gently removed and replaced with 200 μl of ethanol in concentrations of 20, 50, 75, and 90%. After 30 s incubation in RT ethanol was replaced with 100 μl of LB and growth at +37°C was measured in 595 nm wavelength for 20 h in 5 min intervals. In ethanol evaporation experiment, cultures for 96-well plate were prepared as described earlier but volume was lowered into 35 μl per well. Ninety-six-plate cultures were incubated overnight at +37°C without the lid to let the excess media evaporate. Dried biofilms were exposed to 50 μl of ethanol (either 20, 50, 75, or 90%) and left to fully evaporate before addition of 200 μl of LB per well. Growth curve measurements was performed as earlier described.

### Drought Tolerance

The capability to survive over long-lasting drought in a room air humidity was tested by culturing the strains on 96-well plate by transferring overnight grown culture (5 ml LB, +37°C, 210 rpm) in 1:10 ratio to LB. One hundred microliters per well was used with four replicates of each strain. Plates were incubated at +37°C for 3 days in order to grow biofilm. After 3 days, plates were relocated to RT and lids were removed to ensure total evaporation of media. After 12 days, 2 months and 6 months in drought, 200 μl of fresh LB was added into each well and the growth curves were measured at +37°C, 595 nm wavelength, 20 h in 5 min intervals.

### Morphological Characterization

Morphological characteristics of the colonies were analyzed. In order to get single colonies, all strains were cultured in 5 ml LB in +37°C, 210 rpm overnight. Cultures were diluted into 10^−6^ in water and plated onto LB-agar plates. Plates were incubated at +37°C for overnight and the colonies were photographed. Strains EKP5, EKP3 and EKP22 were found to produce translucent colonies along with the traditional colonies. These translucent colonies were further cultivated by transferring one colony to a fresh LB-agar plate and incubated at +37°C overnight or transferred into liquid culture of 5 ml LB and cultured at +37°C and 210 rpm overnight before plating in 10^−6^ dilution onto the new LB-plates. Growth densities were calculated from these plates. From the same liquid culture used for the growth density definition, 1:100 dilutions were made into LB and growth curves were measured in +37°C, 595 nm wavelength, 20 h in 5 min intervals.

### Statistical Analyses

To explore if epidemic or non-epidemic strains can be characterized by their capabilities, we performed discriminant analysis using MASS package (Venables & Ripley, 2002) in R (version. 3.3.2). Effects of individual variables on discriminant function were tested by regressing predicted values against original variables. Overall performance of discriminant function was addressed by Bayesian logistic regression of epidemic status against predicted values of discriminant function using Stan with R (McElreath, 2016).

## Results

### Selection of Strains and Genomic Analysis

**Figure 1:**
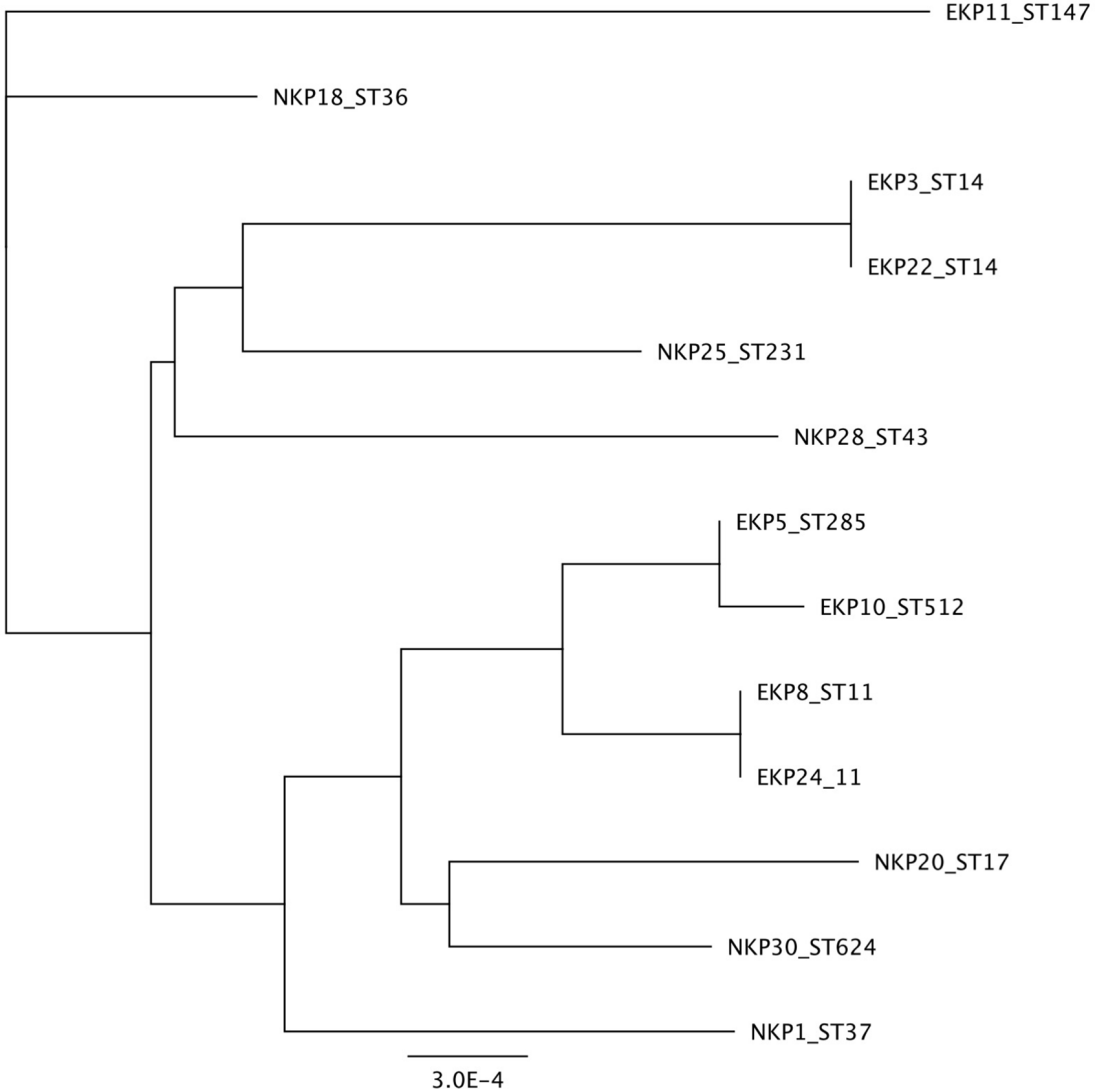
Phylogenetic distance based on Sequence Type sequences.

We selected 14 K*. pneumoniae* strains for detailed phenotypic and genomic analysis in an attempt to identify characteristics that may potentially associate with epidemic STs. The strains were abbreviated either as EKP or NKP for Epidemic and Non-epidemic *K. pneumoniae*, respectively, and the strain number was derived from an internal naming system. The strains and their key genomic traits are listed in **Table 1**. Note that two epidemic STs are represented twice (ST14 and ST11), but their genetic features differ from one another and were thus selected for phenotypic studies in order to evaluate whether the phenotypes of different strains of a single ST are similar. Epidemic and non-epidemic STs are not grouping together when the genomic regions used for sequence typing are used to infer genetic relationship (**Figure 1**). As such, the epidemic strains do not appear to share a common ancestor that diverged from non-successful strains. Therefore, epidemic success is not likely to be linked to a single vertically inherited (genetic) trait, which evolved once. This however does not exclude the possibility that traits preceding epidemic spread are transferred horizontally between strains of different STs. Intriguingly, strains EKP3 and EKP22 (both ST14) and EKP8 and EKP24 (both ST11) are phenotypically different regardless of the same ST (**Supplementary Figure 1**), sometimes being even the opposite phenotypic extremes out of all strains. Colony morphologies of all strains are highly similar (**Supplementary Figure 2**).

The genomes of all the strains were annotated and their overall gene contents compared. Based on the annotation, epidemic and non-epidemic strains appear to be generally uniform metabolically and functionally, hence providing no apparent genome-level design differences to explain epidemic qualities (**Table 2**). Neither the presence or absence of CRISPR system or the number of CRISPR loci appear to be linked with epidemic successfulness. Also, the number of mobile elements such as prophages or plasmids do not associate specifically with either group. The most obvious potentially explanatory features, *i.e.* virulence genes and antibiotic resistance genes, are similar between epidemic and non-epidemic *K. pneumoniae* despite of differences among individual strains (**Tables 1** and **2**). We further speculated that some generally overlooked features such as short open reading frames (ORFs) of length 30 to 150 nucleotides could possibly be linked with epidemic qualities. These genes are rarely identified as coding regions with automated annotation algorithms despite of the fact that they are sometimes transcribed and translated and may reflect recent adaptations to new life strategies or specific conditions (that may be related to epidemic spread). We prepared an algorithm to extract all short ORFs which are preceded by a (near-)perfect ribosome biding site and which do not overlap with existing annotated ORFs (Python code is available in **Supplementary File 1**). On average, approximately 200 unannotated ORFs were extracted from the sequences. Yet, while putative short genes are common, their count is similar in epidemic and non-epidemic strains (data not shown).

**Table 2:** Genomic analysis of individual strains based on RAST-annotation.

	EKP03	EKP05	EKP08	EKP10	EKP11	EKP22	EKP24	NKP01	NKP02	NKP18	NKP20	NKP25	NKP28	NKP30
**Cell Wall and Capsule**	217	230	218	230	234	214	220	216	196	214	217	217	193	227
Capsular and extracellular polysacchrides	38	54	42	54	57	38	41	38	42	36	37	37	39	49
Gram-negative cell wall components	91	90	91	90	90	38	90	89	66	90	88	92	69	92
Cell wall and capsule—no subcategory	88	86	85	86	87	85	89	89	88	87	88	88	85	86
**Virulence, Disease, and Defense**	148	150	141	155	156	154	136	146	143	154	144	168	134	129
Adhesion	7	7	7	7	7	7	7	7	7	7	7	7	7	7
Toxins and superantigens	0	0	0	0	0	0	0	0	0	0	0	0	0	0
Bacteriocins, ribosomally synthesized antibacterial peptides	12	12	12	12	12	12	12	12	12	12	12	12	12	12
Resistance to antibiotics and toxic compounds	121	123	114	128	129	127	109	119	116	127	117	141	107	102
Virulence, disease, and defense	0	0	0	0	0	0	0	0	0	0	0	0	0	0
Detection	0	0	0	0	0	0	0	0	0	0	0	0	0	0
Invasion and intracellular resistance	8	8	8	8	8	8	8	8	8	8	8	8	8	8
**Phages, Prophages, Transposable elements, Plasmids **	47	51	62	57	81	49	44	29	78	78	66	19	77	8
Phage family-specific subsystems	0	0	0	0	0	0	0	0	0	0	0	0	0	0
Transposable elements	0	0	4	0	0	0	0	7	0	0	0	0	5	0
Phages, prophages	47	50	57	57	80	48	43	20	77	77	66	19	70	8
Phages, prophages, transposable elements, plasmids—no subcategory	0	1	1	0	1	1	1	2	1	1	0	0	1	0
Pathogenicity islands	0	0	0	0	0	0	0	0	0	0	0	0	0	0
Gene transfer agent	0	0	0	0	0	0	0	0	0	0	0	0	0	0
Plasmid related functions	0	0	0	0	0	0	0	0	0	0	0	0	0	0
**Membrane Transport**	231	267	214	246	289	346	238	293	247	310	270	325	279	234
Protein secretion system, Type II	19	19	19	19	19	19	19	19	19	19	19	19	19	19
ABC transporters	78	72	65	72	78	79	60	71	68	69	78	71	71	75
Protein secretion system, Type VII (Chaperone/Usher pathway, CU)	20	25	20	25	19	20	25	25	19	20	20	28	26	22
Protein translocation across cytoplasmic membrane	7	7	7	7	7	7	7	7	7	7	7	7	7	7
Protein secretion system, Type V	0	0	0	0	0	0	0	0	0	0	2	0	0	2
Protein secretion system, Type I	5	0	0	0	0	5	0	0	0	5	0	0	0	0
Cation transporters	24	24	22	24	24	24	24	24	23	24	24	25	22	22
Protein secretion system, Type III	0	0	0	0	0	0	0	0	0	0	0	0	0	0
Protein secretion system, Type VI	19	17	16	17	14	19	15	14	0	14	15	14	13	22
Protein secretion system, Type VIII (Extracellular nucleation/precipitation pathway, ENP)	0	0	0	0	0	0	0	0	0	0	0	0	0	0
Protein and nucleoprotein secretion system, Type IV	21	68	28	46	92	137	49	97	73	114	67	125	87	29
**Iron Acquisition and Metabolism**	80	77	67	76	74	80	68	76	74	67	74	70	69	67
Siderophores	18	21	20	20	18	18	20	20	22	18	18	18	17	19
Iron acquisition and metabolism—no subcategory	62	56	47	56	56	62	48	56	52	49	56	52	52	48
Iron transpot	0	0	0	0	0	0	0	0	0	0	0	0	0	0
**Motility and Chemotaxis**	12	10	9	10	11	13	8	10	11	10	11	10	10	10
Magnetotaxis	0	0	0	0	0	0	0	0	0	0	0	0	0	0
Motility and chemotaxis—no subcategory	12	10	9	10	11	13	8	10	11	10	11	10	10	10
Flagellar motility in Prokaryota	0	0	0	0	0	0	0	0	0	0	0	0	0	0
Social motility and nonflagellar swimming in bacteria	0	0	0	0	0	0	0	0	0	0	0	0	0	0
**Regulation and Cell Signaling**	173	167	165	167	168	173	175	178	174	180	170	172	171	174
Quorum sensing and biofilm formation	13	13	13	13	13	13	13	13	13	13	13	13	13	13
Regulation of virulence	9	8	8	8	8	8	8	8	8	8	8	8	8	8
Programmed cell death and toxin-antitoxin systems	16	18	15	17	17	18	23	23	17	19	20	18	19	24
**DNA Metabolism**	155	131	126	140	173	157	145	151	138	144	143	133	142	142
CRISPs	7	0	0	0	7	7	0	0	0	0	0	0	0	7

### Phenotypic Qualities of the Strains

It is possible that epidemic spread selects for or is preceded by specific phenotypic traits. These traits may not necessarily be linked with any particular genetic feature as there may be several mutational pathways to acquire the quality and hence, they may be difficult to identify with genetic or genomic comparisons. As such, we listed a number of measurable phenotypes that may be linked with epidemic success. The traits, their speculated association with epidemic dispersal, and the variables used in this study are listed in **Table 3**. The original data from measurements is available in **Supplementary File 2**. **Supplementary Figure 1** summarizes the results for epidemic and non-epidemic strains.

**Table 3:** Studied characteristics and their hypothesized association with epidemic capability.

Trait/quality	Variables/factors used in this study	Relevance
Virulence genes	Number/type	Number of virulence genes may directly affect the strain’s potential to cause infections
Antibiotic resistances	Number/type	Antibiotic resistance can compromise treatment, thus causing prolonged infections and increase the time during which bacteria disperse
Growth rate/density	Max growth rate (r), Max growth density (K)	Faster and more dense growth may increase the bacterial load in the surrounding environment and thus its epidemic potential
Growth temperature	22°C (room temperature), 37°C	Growth differences in room temperature *vs* 37°C may reflect trade-offs in within- and outside-host environments and thus its adaptation to the environment vs the host
Growth in different media	LB, DMEM, BHI	Potential to grow in different nutrient environments may provide bacteria more opportunities to proliferate in alternative habitats and thus provide possibilities to survive outside the host
Growth in different nutrient levels	1, 10, 100% L	Potential to grow in varying nutrient levels may provide advantage in different environments and hence affect its dispersal to new hosts
Growth in varying pH	pH 3, pH 4, pH 5, pH 6, pH 7	Bacteria may be exposed to different pH in the environment and the host (phagocytosis, skin, gastrointestinal tract) and the sensitivity to pH may decrease the changes for dispersal
Potential to compensate surrounding pH	pH 3, pH 4, pH 5	Potential to modify the surrounding microenvironment may play a crucial role in the bacterial chances to adapt to fluctuating environmental pH and hence affect its dispersal
Resilience in EtOH	20, 50, 75, 90% EtOH	Survival in the presence of alcohol containing sanitizers may directly affect the persistence of the strain in the environment and hence influence its potential to get transmitted
Survival in the absence of water	14 days, 2 months, 6 months	The potential to withstand drought can increase the timespan during which pathogen remains viable in hospital environment and hence affect its changes to get transmitted to new hosts
Recovery after drought	14 days, 2 months, 6 months	Faster recovery after drought can provide bacteria increased potential to colonize or infect new hosts
Growth in mixed/spatially structured population	0 rpm, 230 rpm	Growth as a differently structured population may play a role in various stages of infection and persistence in the environment and may therefore affect the strain’s potential to disperse
Number of plasmids	Number/Inc-type	Plasmids often carry genes that benefit the bacterium in specific conditions and hence their number may be related to survival in various conditions inside and outside the host
Genomic prophages	Number/Inc-type	Activation of prophages may cause infections in competing *K. pneumoniae* strains and may therefore provide the prophage carrying strain an advantage in situations where several strains occupy the same environment
Strain-specific inhibition	Pairwise inhibition	Production of bacteriocins or other antimicrobials may inhibit the growth of competing strains and thus may hinder the potential for sensitive strains to disperse into environments or hosts with other *K. pneumoniae* strains
Short ORFs in the genome	Number of ORFs	Number of short open reading frames in the genome may reflect bacterial adaptive history and hence may be linked to epidemic potential

**Figure 2:**
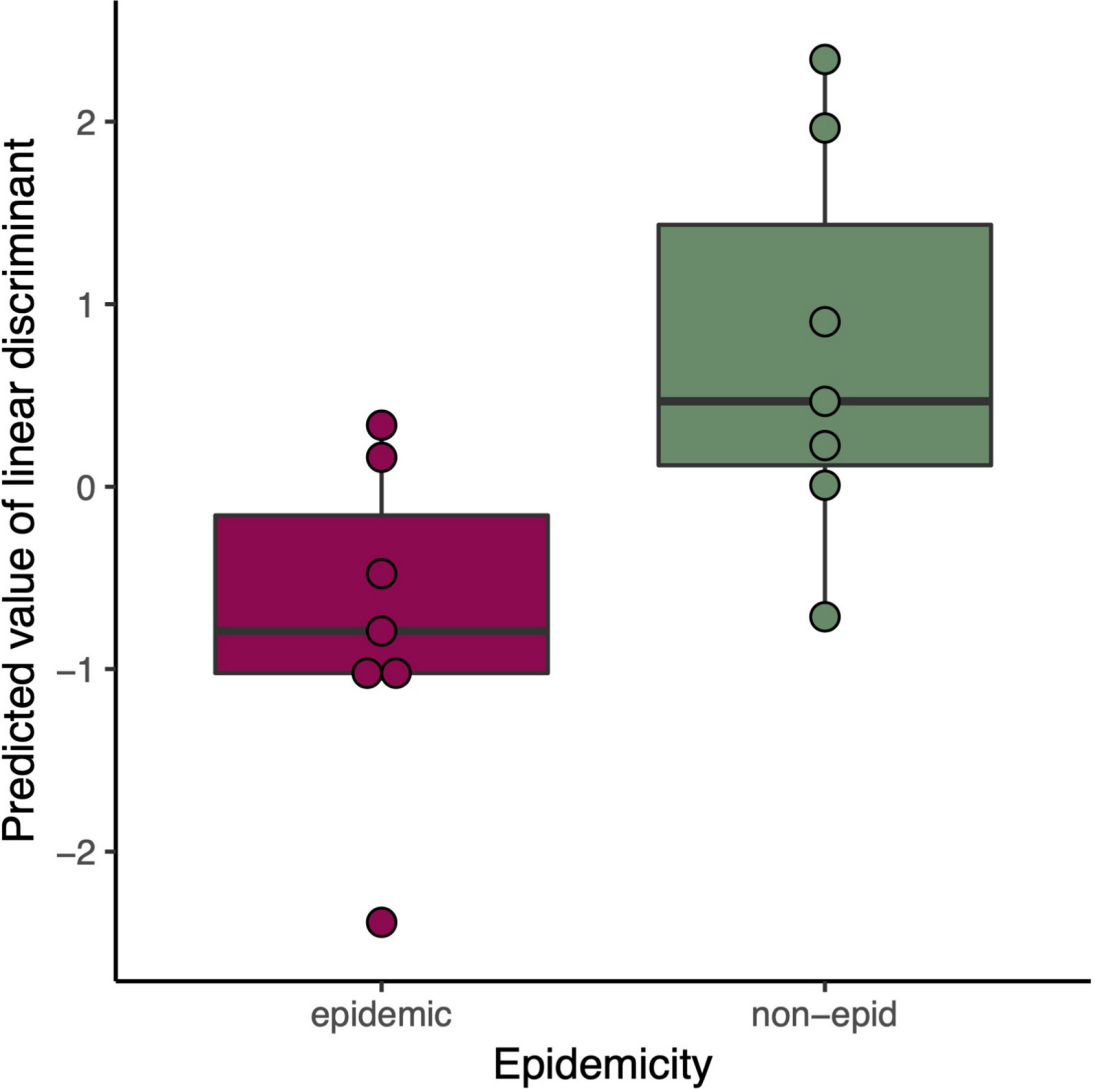
Predicted Linear discriminant values in epidemic (red) and non-epidemic (green) *K. pneumoniae* strains.

**Figure 3:**
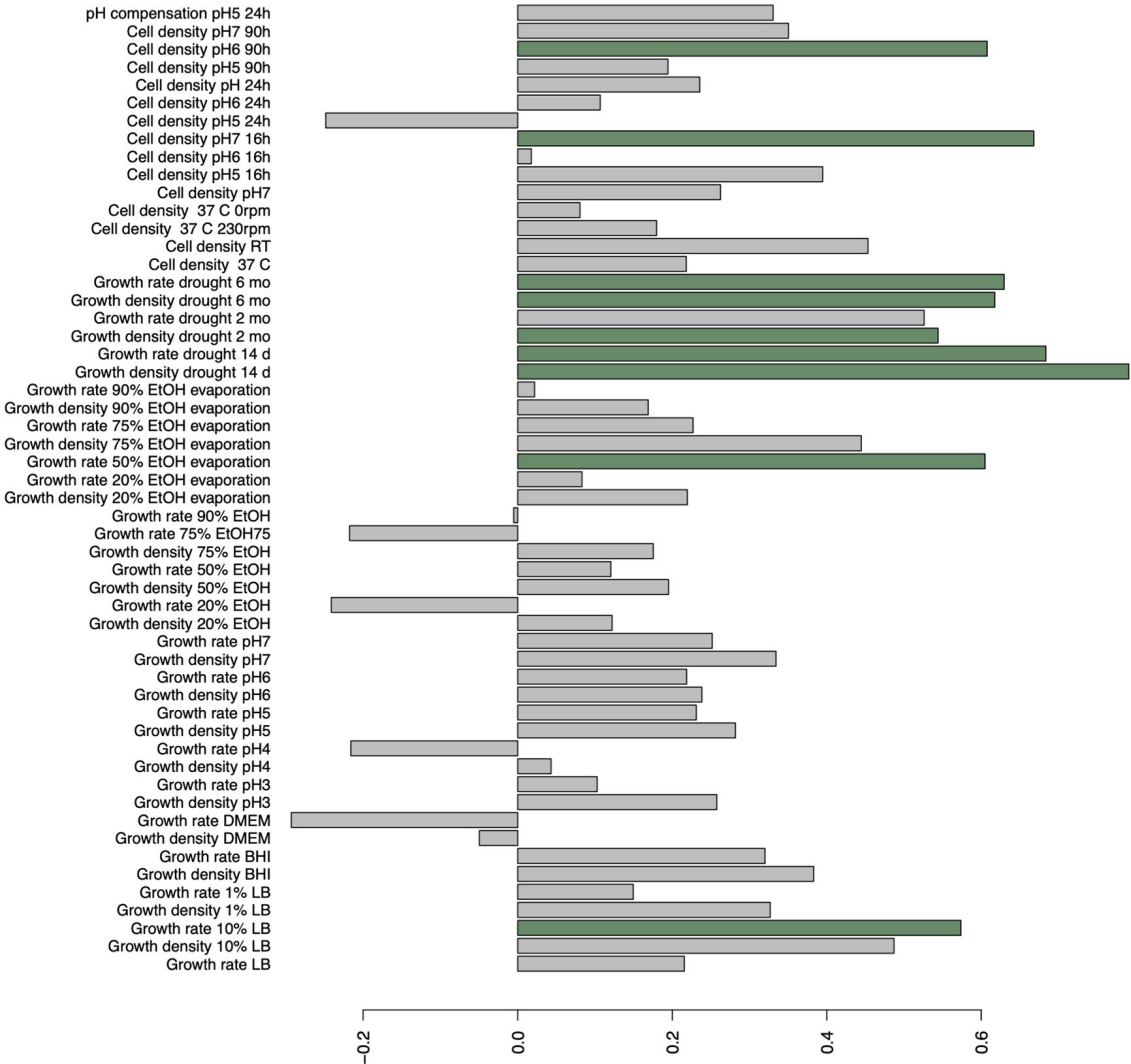
Correlations of original variables on discriminant function explaining the best linear combinations of variables in explaining epidemic status of strains. Bars highlighted with green indicate significant (p < 0.05) correlations.

We carried out a discriminant analysis for the measured phenotypic data. The analysis associated several phenotypic measurements with discriminant function. The discriminant function is a combination of linear effects of variables that give best separation of the data to distinct classes, in this study epidemic and non-epidemic strains. This approach revealed highly significant (posterior values did not overlap with zero) likelihood for a given trait to belong to either epidemic or non-epidemic group (resolved with Bayesian logistic regression between predicted values of discriminant function and epidemic status, Bayesian R_2_ = 45%, **Figure 2**). Regressing predicted values of discriminant function against original variables indicated especially strong role of various measurements of drought tolerance in discriminant function (**Figure 3**) and non-epidemic strains with high discriminant function score were clearly more drought tolerant.

### Cross-Inhibition and Antibacterial Potential of a Putative Colicin

**Figure 4:**
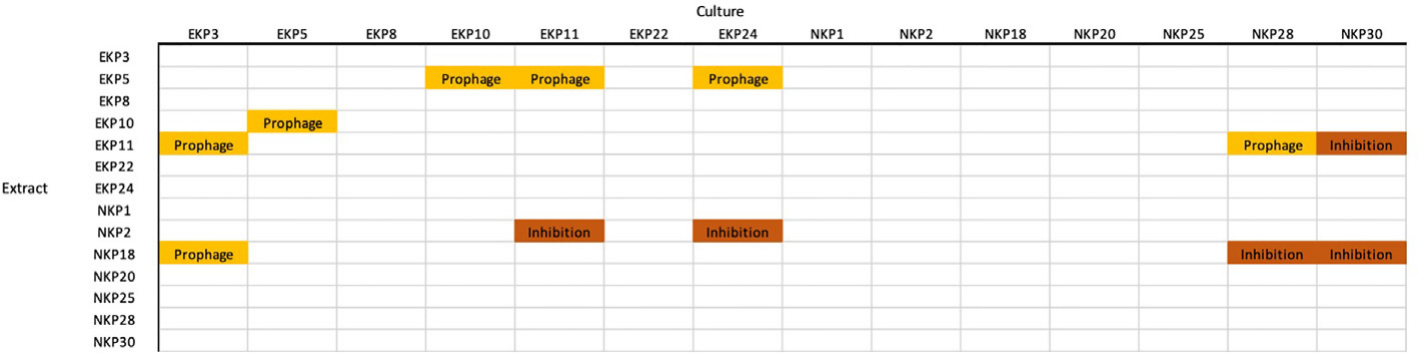
Cross-strain inhibition. “Prophage” indicates that the strain used for generating the extract produces a phage into the medium. “Inhibition” is a non-viral extract that inhibited the growth of the culture strain.

We further studied the cross-strain inhibition given that the epidemic successfulness could emerge from the ability of epidemic strains to suppress non-epidemic strains during spread between hospitals or hosts (**Figure 4**). The inhibition, when detected, was determined with a dilution series to be either due to molecular activity or prophage induction (diluted phages produce distinct plaques unlike inhibiting molecules). Pairwise inhibition was infrequent and no general pattern between epidemic and non-epidemic strains was identifiable.

As a curiosity, we selected one cross-inhibiting strain pair for more detailed analysis. NKP2 produces a component into its medium that inhibits EKP24 (ST11). Given the wide dispersal of ST11 *K. pneumoniae* and its association with NDM-1 encoding plasmids and hypervirulence (Gu et al., 2017), the inhibiting factor could provide a possible way to control these strains. However, the strain used in this study is not hypervirulent and therefore assessing direct applicability against hypervirulent strains was not conducted. Genomic comparison of NKP2 and EKP24 revealed the presence of genes for Colicin E3 in NKP2 that were absent from EKP24. Hence, Colicin E3 was hypothesized to be the inhibiting molecule. Co-culturing of these strains in the same medium demonstrates that EKP24 is unable to multiply. We further studied the adaptation of EKP24 to the continuous presence of the hypothesized Colicin E3 by serially culturing EKP24 in the presence of NKP2 medium extract for 4 weeks (n = 5). These cultures were refreshed three times a week. After 2 weeks, we removed the selection from five additional replicates. Resistance to NKP2 medium (with hypothesized Colicin E3) emerged already during the first culture transfer, and it remained stable even after the removal of selection. Re-sequencing of three putative Colicin E3 resistant samples revealed a prevalent mutation in Aerobactin siderophore receptor IutA. This mutation was absent in a culture that was not exposed to NKP2 extract. IutA has been shown to serve as a receptor for cloacin DF13 (Van Tiel-Menkveld et al., 1982), which is homologous to Colicin E6 and E3 (Akutsu et al., 1989). As such, we hypothesize that NKP2 extract rapidly selected for IutA mutants. Also, it is worth noting that NKP2 medium was not observed to inhibit the growth of the other ST11 strain EKP8, hence showing narrow activity. Altogether, the hypothesized Colicin E3 does not appear to provide efficient antimicrobial activity against drug-resistant *K. pneumoniae* strains even when the targeted strain is initially sensitive to the colicin.

## Discussion

*K. pneumoniae* has become one of the priority drug-resistant pathogens in hospital settings worldwide. Some *K. pneumoniae* STs are more prevalent compared to others, which provokes the question whether there are qualities in these genetically related groups that have made them more potent for dispersal. Here, we studied multiple phenotypic and genotypic characteristics of seven epidemic and non-epidemic carbapenem resistant *K. pneumoniae* isolates that emerged from different parts of the world. None of the specific genetic qualities associated uniformly with epidemic or non-epidemic strains. Overall, this again suggests that sequence typing is not an optimal approach for inferring qualities of individual pathogens. In other words, there are *K. pneumoniae* strains that are relatively different from one another for their specific characteristics while still grouping together in ST-analyses. Phenotypic characterization revealed indication that, while genetic differences were minute based on ST-analyses, phenotypic differences that separate epidemic and non-epidemic *K. pneumoniae* strains do exist. Such apparently controversial result could emerge if phenotypes are strongly dictated by genetic differences other than indicated by core genome-based ST-analyses or even by epigenetic modifications (Casadesus and Low, 2006).

While most of the studied factors could not explicitly help explain epidemic qualities, the strong association of drought tolerance with epidemically non-successful strains could give some insights on the dissemination. All the isolates in this study were able to withstand 6 months of dryness. Interestingly, non-epidemic strains generally recovered faster and into higher density after the drought, which presents the possibility that non-epidemic strains may have qualities that provide them opportunities to cause infections in specific cases, for example, after long-term residence on surfaces (Kramer et al., 2006). Bacteria have multiple ways to protect itself during the drought. For example, effective biofilm formation is a major protection mechanism in bacterial species incapable of endosporulation (Sunde et al., 2009; Reza et al., 2019) or cyst formation (Malinich and Bauer, 2018). Nosocomial carbapenem resistant *K. pneumoniae* infections are mainly acquired by two routes, either in person-to-person contact (Casewell and Phillips, 1977) or *via* contaminated surfaces and instrumentation (Jarvis et al., 1985; Revdiwala et al., 2012). Also, a gut carriage of carbapenem resistant *Klebsiella* have been associated with an elevated infection risk (Tischendorf et al., 2016; Gorrie et al., 2017). It is possible that drought tolerant strains are more likely to remain viable on inanimate surfaces, from which they are only rarely transferred to the patient. On the other hand, the epidemically successful strains could be more adapted to human host and tolerated as the colonizing pathogens. These might be two different strategies for the same species to survive in the shared environment and results of evolutionary trade-offs between inside host and outside host conditions (*e.g.* Mikonranta et al., 2015; Ashrafi et al., 2018). However, the reasons why certain STs seem to develop higher tolerance towards dryness remains unclear.

*K. pneumoniae* has also noted to be able to grow in acidic pH and even capable to compensate acidic environment of phagosomes (Cano et al., 2015). All the strains in this study tolerated decreasing pH up to 4. In pH 3 no viable dividing cells were detected and for that reason no pH compensation was observed either. In the viable cultures with initial pH of 4, cells were able to increase the pH in the surrounding media by 1.04–1.62 in 24 h. Strain EKP10 was an exception as it was barely able to grow in pH 4 and could not compensate the low pH. Altogether, long-term exposure to environments that have pH of 3 or below serves as a potential barrier for *K. pneumoniae* viability. Increasing tolerance towards the alcohol-based hand rubs and sanitizers have been observed in multiple species of XDR bacteria. It has been suggested as one possible explanation to the increasing emergence of these pathogens in hospital environments (Pidot et al., 2018). The *K. pneumoniae* isolates utilized in this study, however, were not able to withstand ethanol at concentrations 50% (v/v) or above.

Overall, different *K. pneumoniae* strains vary in their phenotypic characteristics and hence may cause infections *via* differing routes. This highlights the importance of exerting precautionary steps in healthcare. Namely, these include early recognition of both carriers and infected patients (Borer et al., 2012; Gorrie et al., 2017; Liu et al., 2019), and controlling the spread of pathogens with apparently still effective hand hygiene and disinfection of inanimate surfaces (Kramer et al., 2006; Borer et al., 2012; Gorrie et al., 2017). Multiple studies also focus on novel approaches to prevent infections by coating endotracheal tubes and catheters with new antibacterial agents (Caratto et al., 2017; Bjorling et al., 2018; Seitz et al., 2019). Such multifrontal measures to control *K. pneumoniae* is likely to be necessary for tempering both epidemic and non-epidemic strains.

In conclusion, we were able to detect one specific quality that associated with epidemic status of *K. pneumoniae* isolates. Drought tolerance and recovery from dryness of up to 6 months significantly associated with the non-epidemic strains. Thus, we need to consider the possibility that non-epidemic strains may have unique advantages in specific conditions that let them occasionally show up as local epidemics in surveillance studies. Additionally, some non-epidemic isolates inhibited the growth of epidemic strains. This may allow these strains to cause infections instead of their epidemic contemporaries. However, it is possible although unlikely that pure coincidence may be behind the epidemic success of certain *K. pneumoniae* STs. The initial establishment, instead of any specific characteristic, may have provided certain strains chances to acquire new virulence factors and resistance elements, hence furthering their spread.

## Data Availability Statement

All data is available either as **Supplementary Material** or in GenBank.

## Author Contributions

KK and MJ conceived and planned the experiments. KK, RP, and MJ carried out the experiments. KK assembled and curated the results. MJ prepared the script for finding ORFs. TK conducted statistical analyses. A-MÖ-G and CG collected the bacterial strains. KK, MJ, RP, and A-MÖ-G conducted genetic analyses. All authors contributed to the interpretation of the results. First draft of the manuscript was written by KK and MJ and other authors provided critical feedback and helped shape the research, analysis, and the final manuscript. All authors contributed to the article and approved the submitted version.

## Funding

Authors wish to acknowledge funding from the Academy of Finland (grants no.252411, no.297049 and no. 336518 to MJ and no. 322204 to RP), Emil Aaltonen Foundation and Jane and Aatos Erkko Foundation.

## Conflict of Interest

The authors declare that the research was conducted in the absence of any commercial or financial relationships that could be construed as a potential conflict of interest.

## Supplementary Material

Supplementary Figure 1 Summary of all measurements.

Supplementary File 1 Phyton script for short ORFs.

Supplementary File 2 Original measurement data.
