# Supplementary material for: Systematic Comparison of Epidemic and Non-Epidemic Carbapenem Resistant Klebsiella pneumoniae Strains
Source: Front Cell Infect Microbiol. 2021 Feb 23;11:599924. doi: 10.3389/fcimb.2021.599924 (PMC7940544; doi:10.3389/fcimb.2021.599924)
Supplement: Supplementary Figure 1 — Summary of all measurements. [file DataSheet_3.zip › Supplementary Figure 2.docx]

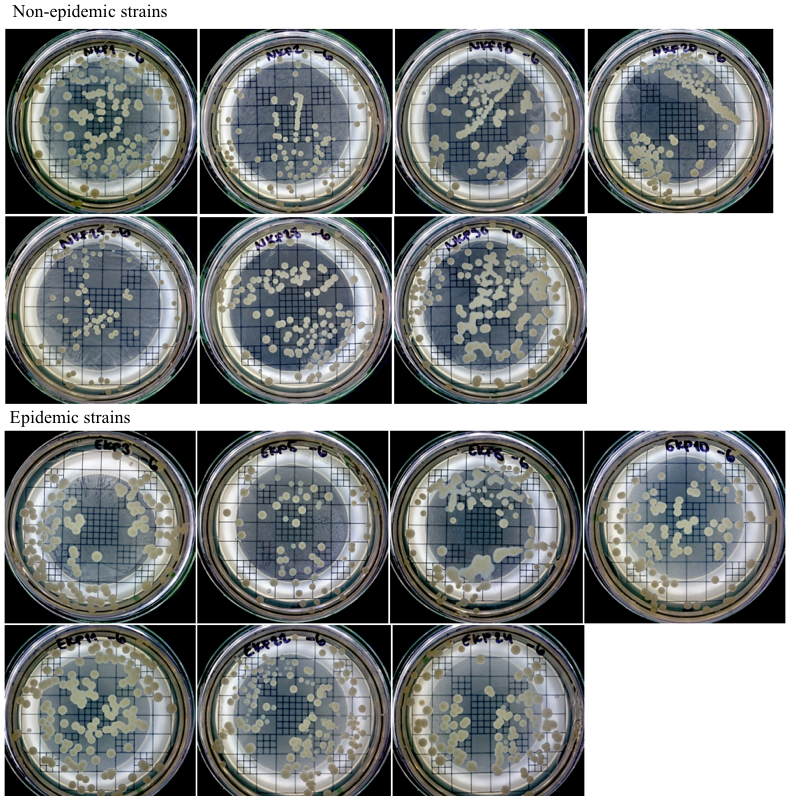


**Supplementary Figure 2**. Colony morphologies of non-epidemic and epidemic *K. pneumoniae* strains used in the study.
