# Supplementary material for: Systematic Comparison of Epidemic and Non-Epidemic Carbapenem Resistant Klebsiella pneumoniae Strains
Source: Front Cell Infect Microbiol. 2021 Feb 23;11:599924. doi: 10.3389/fcimb.2021.599924 (PMC7940544; doi:10.3389/fcimb.2021.599924)
Supplement: Supplementary File 1 — Phyton script for short ORFs. [file DataSheet_1.pdf]

```
import re
import shutil
import os
from Bio.Seq import Seq


def remove_overlaps_with_existing_orfs(lenreverse, is_reverse,
id_nro, tallennus, start,end,sekvenssi, fasta, dir):
    for filu in os.listdir(dir):
        if filu.endswith(".bed"):
            bedi = open(filu)
    fasta = fasta.replace(" ", "_")
    idsa = id_nro
    k = 0;
    s = 0;

    testi = False
    if os.path.exists(dir+"/"+tallennus):
        orfit=open(dir+"/"+tallennus+"_short_ORFs.fas", "w")
    else:
        orfit=open(dir+"/"+tallennus+"_short_ORFs.fas", "a")

    for line in bedi:

        columns=line.rstrip("\n").split('\t')
        if len(columns) > 1:


            columns[0] = columns[0].replace(" ", "_")
            if columns[0] == fasta:
                if not re.search(r"source", columns[3]):
                     if re.search(r"CDS", columns[3]):
                         testi = True

                         alku = int(columns[1])
                         loppu = int(columns[2])
                         if not is_reverse:
                            if ((start >= alku) and (start<=loppu))
or ((end>=alku) and (end<=loppu)):
                                k=k+1;
                         if is_reverse:
                             loppu_rev = lenreverse-alku

                             alku_rev =lenreverse-loppu

                             if ((start >= alku_rev) and
(start<=loppu_rev)) or ((end>=alku_rev) and (end<=loppu_rev)):
```

```
                    k=k+1;



    if (k == 0 and testi):
        idsa = idsa+1
        orfit.write(">"+tallennus+"_ORF"+str(idsa)+"\n")
        orfit.write(sekvenssi+"\n")
    orfit.close()
    return idsa

dire = ”PATH/Python_test_bed/"
os.chdir(”PATH/Python_test_bed/")
stop_codons = ["TGA", "TAA", "TAG"]
for file_name in os.listdir(os.getcwd()):
    ids = 0
    os.chdir(”PATH/Python_test_bed/")
    if os.path.isdir(file_name):
        print(file_name)
        print(os.getcwd())
        os.chdir(”PATH/Python_test_bed/"+file_name+"/")
        print(os.getcwd())
        for filet in os.listdir(os.getcwd()):
            print(filet)
            if filet.endswith(".fasta"):
                print(file_name)
                data = open(os.getcwd()+"/"+filet)
                fasta_id = ""
                jou = []
                l= 0
                for line in data:
                    rivi=line.rstrip("\n")
                    if rivi[0] == ">":
                        fasta_id = rivi[1:]
                        print(fasta_id)
                    else:
                        seq = Seq(rivi)
                        rev = seq.reverse_complement()
                        m = re.finditer(r"(?=(C|A|G)(A|T)G(G|A)A(A|
G|T)(A|G|T)[ATGC]{2,7}?)ATG(?:[ATGC]{3}){30,150}?(?:TAG|TAA|TGA)",
rivi)
                        for match in m:
                            te = lambda x : any(x[i:i+3] in
stop_codons for i in range(0,len(x)-3,3))
```

```
                                    if (te(match.group()) == False):

                                            ids =
remove_overlaps_with_existing_orfs(0, False, ids, file_name,
match.end(), match.start(), match.group(), fasta_id, os.getcwd())

                                n = re.finditer(r"(?=(C|A|G)(A|T)G(G|A)A(A|
G|T)(A|G|T)[ATGC]{2,7}?)ATG(?:[ATGC]{3}){30,150}?(?:TAG|TAA|TGA)",
str(rev))
                                for match in n:
                                    te = lambda x : any(x[i:i+3] in
stop_codons for i in range(0,len(x)-3,3))

                                    if (te(match.group()) == False):

                                            ids =
remove_overlaps_with_existing_orfs(len(rev), True, ids, file_name,
match.end(), match.start(), match.group(), fasta_id, os.getcwd())
```
